# Davanloo’s Intensive Short-Term Dynamic Psychotherapy in a tertiary psychotherapy service: overall effectiveness and association between unlocking the unconscious and outcome

**DOI:** 10.7717/peerj.548

**Published:** 2014-08-28

**Authors:** Robert Johansson, Joel M. Town, Allan Abbass

**Affiliations:** 1 Centre for Emotions and Health, Dalhousie University, Halifax, Nova Scotia, Canada; 2 Department of Behavioural Sciences and Learning, Linköping University, Linköping, Sweden

**Keywords:** Psychotherapy, Psychodynamic psychotherapy, ISTDP, Effectiveness, Affect, Emotion

## Abstract

**Background.** Intensive Short-Term Dynamic Psychotherapy (ISTDP), as developed by Habib Davanloo, is an intensive emotion-focused psychodynamic therapy with an explicit focus on handling resistance in treatment. A core assumption in ISTDP is that psychotherapeutic effects are dependent on in-session emotional processing in the form of rise in complex transference feelings that occurs when treatment resistance is challenged. Recent research indicates that an unlocking of the unconscious, a powerful emotional breakthrough achieved at a high rise in complex transference feelings, can potentially enhance ISTDP’s effectiveness. While ISTDP has a growing evidence base, most of the research conducted has used small samples and has tested therapy delivered by expert therapists. The aims of this study were to evaluate the overall effectiveness of ISTDP when delivered in a tertiary psychotherapy service, and to investigate if having an unlocking of the unconscious during therapy predicted enhanced treatment effectiveness.

**Methods.** A total of 412 patients were included in the analyses. The average length of treatment was 10.2 sessions (SD 13.3). Multilevel growth curve modeling was used to evaluate treatment effectiveness and the association between unlocking the unconscious and outcome. A number of control predictors including type of treatment resistance were selected and included in the analyses. Outcome measures were the Brief Symptom Inventory (BSI) and the Inventory of Interpersonal Problems (IIP). About half of the patients in the study were treated by therapists in training and the other half by more experienced therapists.

**Results.** Growth curve analyses using the full intention-to-treat sample revealed significant within-group effects of ISTDP on both the BSI and the IIP. Effect sizes were large (>0.80). Unlocking the unconscious during therapy was associated with significantly larger treatment outcome. The relationship was further moderated by type of treatment resistance.

**Conclusion.** This study adds to the empirical base of Davanloo’s ISTDP with confirmed treatment effectiveness in a large-scale patient sample when ISTDP was delivered by therapists with a range of experience. Furthermore, emotional mobilization in the form of unlocking the unconscious was confirmed as a process factor enhancing the effectiveness of ISTDP.

## Introduction

Mental disorders are common conditions that generate huge costs for society and cause a great deal of suffering for the affected individuals and their families (Ustün, 1999; Kessler et al., 2007). Several mental conditions such as depression, anxiety, personality disorders and a range of medically unexplained symptoms are suggested to be linked to *adverse childhood experiences* (ACE; Felitti et al., 1998; Edwards et al., 2003; Anda et al., 2006). Problems arising from ACE are suggested to be byproducts of strong unprocessed emotions coupled with deficits in capacity to regulate emotions (Schore, 2001; Schore, 2002; Anda et al., 2006). Research indicates that several individuals with conditions linked to ACE are likely to be treatment-resistant or to experience recurrences (Chartier, Walker & Naimark, 2010). One consequence of this treatment resistance can be chronic illness and large system costs (Nanni, Uher & Danese, 2012). Recently, there have been calls for the development and evaluation of intensive and alternative treatments that address treatment resistance in these patient populations (Nanni, Uher & Danese, 2012).

Intensive Short-Term Dynamic Psychotherapy (ISTDP; Davanloo, 1990; Davanloo, 2000; Davanloo, 2005; Abbass, Town & Driessen, 2012) is an emotion-focused psychodynamic psychotherapy that explicitly addresses treatment resistance in somatic conditions, mood, anxiety and personality disorders arising from ACE. Psychotherapeutic effects in ISTDP are hypothesized to be dependent on in-session mobilization of emotional processes in the form of *rise in complex transference feelings*, defined by co-occurring increases in treatment resistance, unconscious anxiety, therapeutic alliance and experience of feelings (Davanloo, 2005). A *major unlocking of the unconscious* is a powerful in-session emotional breakthrough achieved at a high rise in complex transference feelings assumed to take place when treatment resistance has been adequately challenged. During an unlocking of the unconscious the patient experiences intense complex feelings towards the therapist or another current figure, in which the experience is linked to feelings to past figures and emotion-laden memories about painful feelings, situations and events from the past, i.e., events related to adverse childhood experiences. A fundamental assumption in Davanloo’s ISTDP is that the unlocking of the unconscious is associated with therapeutic gains. Recent research from a sample of 89 patients, all treated by an experienced ISTDP therapist, support Davanloo’s claim (Town, Abbass & Bernier, 2013). In addition, ISTDP has been shown to be effective for a range of conditions (Abbass, Town & Driessen, 2012; Town & Driessen, 2013) including patients who failed to respond to specialist medical, surgical or psychiatric care (Baldoni, Baldaro & Trombini, 1995; Abbass, 2006; Abbass, Lovas & Purdy, 2008; Abbass, Town & Bernier, 2013), or who used an unusually high amount of emergency services (Abbass et al., 2009). However, a majority of existing studies have included small samples and therapies delivered by expert therapists, and are thereby limited in generalizability (Abbass, Town & Driessen, 2012).

The major aims of this study were to evaluate the effectiveness of ISTDP in a tertiary psychotherapy service where patients had been referred from specialist care, and to study if unlocking the unconscious was associated with outcome after controlling for potentially confounding variables, including treatment resistance. The present study differs from most previous research on ISTDP in that it is based on a large clinically representative sample of patients treated by therapists with a range of experience and that it uses multilevel growth curve techniques to model symptom change over time.

## Materials & Methods

This study is reported in accordance with the CONSORT statement for clinical trials (Schulz, Altman & Moher, 2010). The study is an evaluation of the effectiveness of a treatment given in a tertiary care clinic and used anonymized data collected as part of standard care. The project was reviewed and approved by the Capital District Health Authority Research Ethics Board in Halifax, Nova Scotia (approval number 2007-050), and is registered in Clinicaltrials.gov as identifier number NCT01924715.

### Setting for treatment delivery

All patients received treatment at the Centre for Emotions and Health located in Halifax, Nova Scotia, Canada. The centre is a tertiary psychotherapeutic service linked to Dalhousie University and located in the Queen Elizabeth II Health Science Centre in Halifax. Furthermore, the centre is a teaching and research service specializing in assessing and treating emotional contributors to medically unexplained symptoms, anxiety, depression and personality disorders using ISTDP.

### Participants and procedure

**Figure 1:**
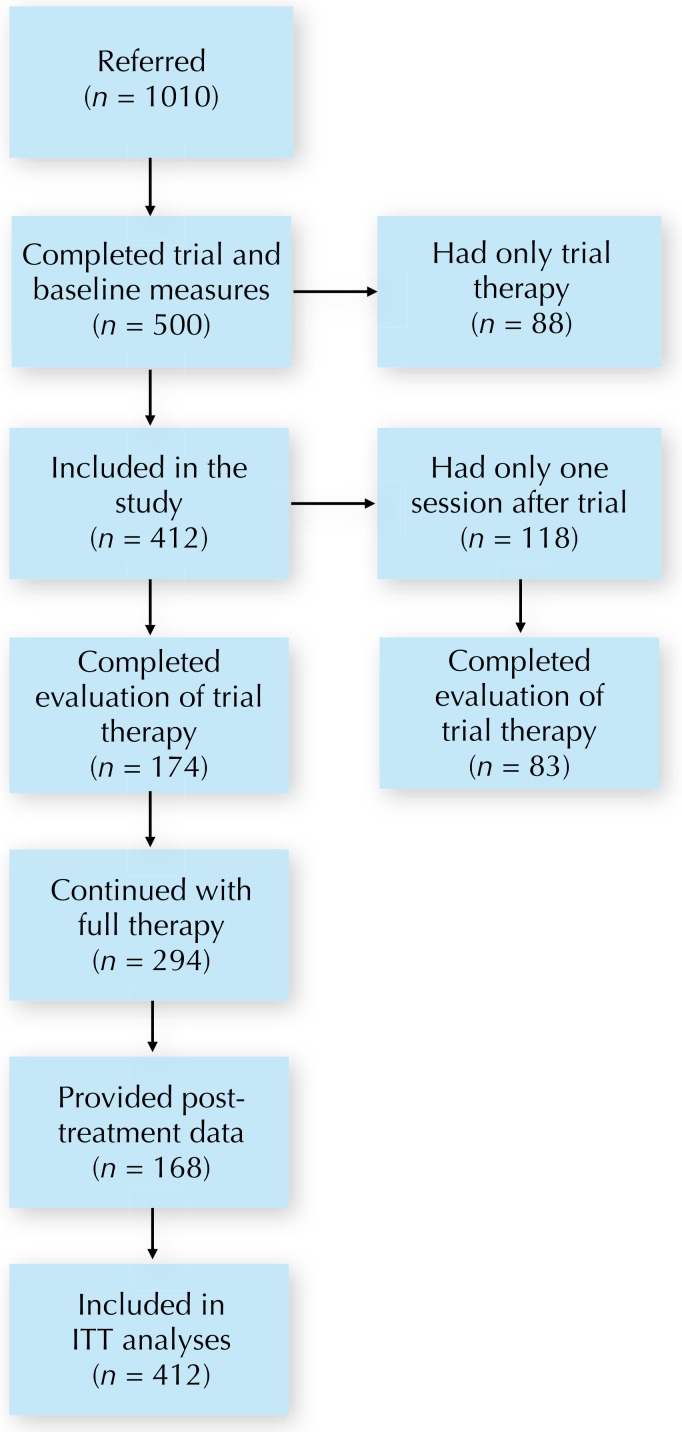
CONSORT flowchart. Abbreviations: ITT, intention-to-treat.

Participants in the study were collected from a large sample of patients referred to the Centre for Emotions and Health between March 30, 1999 and March 30, 2007. A substantial amount of patients referred were seen for an assessment meeting only. In this study, we investigated outcome for all patients who received at least one session of ISTDP after the initial assessment session (the trial therapy). As routine self-reported outcome measures were only implemented part way through this 8 year period, only a portion of the entire treated sample could be included in the effectiveness evaluation. Hence, a further criteria for inclusion in this study was that the patient had a baseline measurement of self-reported symptom distress (see below). The procedure described below is illustrated in the CONSORT flowchart in Fig. 1. All participants in the study were referred by professionals from various specialties, including emergency department, family practice offices, medical-surgery and mental health. Patients referred to the Centre were placed on a wait-list for a trial of therapy, i.e., an initial assessment session of ISTDP. During trial therapy a psychodiagnostic evaluation was conducted which included an assessment of type and degree of treatment resistance according to specified criteria. In addition, baseline assessments of self-rated symptom distress and interpersonal problems (regarding the previous two week period) were collected at the end of the trial session, at the end of the session following the trial, and at treatment termination. Treatment was not time-limited. Rather, termination was determined by response to treatment and agreed upon by patient and therapist.

### Intervention and therapists

#### Intensive Short-Term Dynamic Psychotherapy

The treatment used in this study was Intensive Short-Term Dynamic Psychotherapy (ISTDP; Davanloo, 1990; Davanloo, 2000; Davanloo, 2005; Abbass, Town & Driessen, 2012). ISTDP is a brief psychotherapy based on traditional psychodynamic principles, however, with a strong focus on emotional mobilization and handling of in-session defenses against experiencing of emotions (i.e., treatment resistance). Importantly, experiencing emotions can include a range of experiences including emotional closeness with the therapist. The first session in ISTDP, the trial therapy, is typically longer than the following sessions and involves a psychodiagnostic evaluation, i.e., a thorough assessment of problems in relation to emotional experience, how and to what degree the patient defends against emotions in session and the patient’s capacity to tolerate anxiety (Abbass, Joffres & Ogrodniczuk, 2008). The trial therapy also involves emotional experience of repressed feelings related to ACE where possible. For a substantial amount of patients, the trial on its own brings symptom relief and eliminates the need for further therapy (Abbass, Joffres & Ogrodniczuk, 2008).

#### ISTDP and treatment resistance

ISTDP tailors the treatment process to different patient categories based on patients’ capacity to tolerate anxiety and work through emotions as they arise. Davanloo (2001) has defined two spectra that help characterize patient functioning according to degree and type of resistance. *Psychoneurotic* patients are patients with an intact psychic structure with formal defenses they use in session. These patients do not experience cognitive perceptual disruption and do not rely on primitive defenses such as projective identification. *Fragile* patients, on the other hand, have unconscious anxiety manifest as cognitive perceptual disruption (e.g., dissociation) and have access to primitive defenses at either a low, moderate or high level of emotional activation (Davanloo, 2001). Such a pattern implies that these patients have a less intact defensive structure. Hence, this is another type of treatment resistance. ISTDP for psychoneurotic and fragile patients tends to be different. For psychoneurotic patients, where patients can better tolerate emotional experiences, defenses towards such experiences are explicitly challenged. Fragile patients require process to build capacity to tolerate anxiety and emotions (Davanloo, 1990; Whittemore, 1996; Abbass & Bechard, 2007). This latter format also applies to patients with severe depression and somatic conditions such as conversion and irritable bowel syndrome (Davanloo, 2005). Patients with psychotic disorders can also benefit from this capacity-building format of ISTDP (A Abbass, D Bernier, S Kisely, JM Town & R Johansson, 2014, unpublished data; Abbass, 2002). When working with ISTDP for psychotic patients the therapy is supportive and devoid of any challenge to defenses. The therapy is facilitative of an emotionally active learning where unconscious processes are studied and underscored toward mastering emotions and developing better tolerance of anxiety. Thereafter, some of these unprocessed emotions may be experienced to facilitate grieving of losses and resolution of internal conflicts. Losses related to the illness and emotions around imposed treatments and hospitalizations are also grieved (A Abbass, D Bernier, S Kisely, JM Town & R Johansson, 2014, unpublished data).

#### Therapists

Therapists were licensed health professionals and trainees learning ISTDP. One of the therapists was a highly experienced ISTDP trainer and supervisor, considered an expert in the field. All therapists were part of weekly small-group supervision led by the experienced ISTDP trainer. Supervision included review of video recordings of treatment sessions (Abbass, 2004). Furthermore, the therapists were provided technical literature on ISTDP and attended weekly didactic courses.

### Outcome measures

Treatment effectiveness was evaluated using the Brief Symptom Inventory (BSI; Derogatis & Melisaratos, 1983) and the Inventory of Interpersonal Problems, 32 item version (IIP; Horowitz et al., 1988). The Global Severity Index (GSI), derived using the procedures from the BSI manual, was used as a measure of general symptom distress. For the IIP, the total mean score was used as an overall measure of problems in relationships related to, for example, self sacrificing and lack of assertiveness. Hence, this measures problems that are assumed to be ameliorable with ISTDP. These measures were administered at baseline, at the first meeting after the trial therapy session, and at treatment termination.

### Rise in complex transference feelings and treatment resistance

Within the framework of ISTDP, distinguishable phases that occur during mobilization of unconscious processes have been established (Davanloo, 1990). These phases, the degree of *rise in complex transference feelings*, aim to distinguish phases of mobilization of unconscious emotional processes, defined by co-occurring increases in resistance, unconscious anxiety, the experience of feelings and the unconscious therapeutic alliance. According to Davanloo (2005), the phases have been replicated through repeated case series data. The *major unlocking of the unconscious* is a state possible to reach at a high rise in complex transference feelings (i.e., with a strong mobilization of unconscious processes) after resistance has been systematically challenged. This state is defined as a passage of intense complex feelings towards the therapist (or another current figure), in which a past person’s image is transferred onto the current figure. ISTDP suggests this to be a passage of complex feelings towards the past figure triggered by the complex feelings activated in the therapy session. Typically this experience leads to emotion-laden memories about painful feelings, situations and events from the past, i.e., events related to adverse childhood experiences. In this state, the patient typically displays no resistance in the process and has far less anxiety. Thereafter, the process of linking in-session resistance and avoided feelings to past adverse experiences can proceed with few obstacles (Davanloo, 2005; Town, Abbass & Bernier, 2013).

For ratings of rise in complex transference feelings in this study, we used a quantitative coding system with categories as follows: (1) low rise in complex transference feelings, (2) high rise in complex transference feelings, (3) partial unlocking of the unconscious, (4) major unlocking of the unconscious, and (5) extended major unlocking of the unconscious. Details of these phases can be found in Davanloo’s writings (Davanloo, 2001; Davanloo, 2005). As one of the primary aims of this study was to investigate whether having a major unlocking of the unconscious (categories 4 and 5) predicted treatment outcome (as suggested by Town, Abbass & Bernier (2013)), we included a binary variable that captured this (1 = “did have a major unlocking of the unconscious during therapy”, 0 = “did not have a major unlocking of the unconscious during therapy”).

According to Davanloo (1990), Davanloo (2001) and Davanloo (2005), rise in complex transference feelings are dependent on the *type and degree of patient resistance* operating at any given point during the psychotherapeutic encounter. Patient resistance is defined as any unconscious or previously unconscious defense operating in the therapy relationship. Based on case-series data from several hundred patients, Davanloo (2001) and Davanloo (2005) established operationally defined patient categories of treatment resistance based on the observation of in-session use of defenses and anxiety discharge patterns. As described above, a patient is considered *fragile* when primitive defenses and cognitive perceptual disruption are prominent features and *psychoneurotic* when not. This is the basis for how Davanloo (1990) established two types of resistance that each is a *spectrum* of degree of resistance. Davanloo (2001) named these the *spectrum of patients with psychoneurotic disorders* and the *spectrum of patients with fragile character structure*. In this particular study, we used a coding of treatment resistance as follows: (1) low resistance, (2) moderate resistance, (3) high resistance, (4) fragile, and (5) psychotic. As the purpose of this particular study was to control for type of defensive structure, we included a binary variable that described this (0 = “low to high resistance”, 1 = “fragile or psychotic”).

Coding of treatment resistance and rise in complex transference feelings was conducted during supervision by the experienced ISTDP supervisor using review of video recordings of treatment.

### Statistical analyses

The study had an open design in that no control group was used. A set of analyses were carried out that focused both on the within-group effects of treatment on outcome (time effects) and on whether having an unlocking of the unconscious during therapy predicted these effects. These analyses were addressed with growth curve modeling using SPSS version 21 (SPSS, Inc., Chicago, IL) and followed the procedures described by Singer & Willett (2003).

A series of growth curve models of the trajectories of the BSI and the IIP were generated and tested. First, unconditional growth models were estimated for both variables to examine the average growth over the course of treatment.

The equations for the unconditional growth models estimating the effects of therapy over time were as follows:

Level-1 Model:

\begin{eqnarray*} \displaystyle {Y}_{i j}={\pi {}_{0}}_{i}+{\pi {}_{1}}_{i}\times {T I M E}_{i j}+{\varepsilon }_{i j}.&&\displaystyle \end{eqnarray*}

Level-2 Model:

\begin{eqnarray*} \displaystyle \mathrm{Intercept}{:}~{\pi {}_{0}}_{i}={\gamma }_{00}+{\zeta \! {}_{0}}_{i}&&\displaystyle \nonumber\\ \displaystyle \mathrm{Slope}{:}~{\pi {}_{1}}_{i}={\gamma }_{10}+{\zeta \! {}_{1}}_{i}&&\displaystyle \nonumber\\ \displaystyle {\varepsilon }_{i j}\sim N(0,{\sigma }_{\varepsilon }^{2}),{\zeta \! {}_{0}}_{i}\sim N(0,{\sigma }_{0}^{2}),{\zeta \! {}_{1}}_{i}\sim N(0,{\sigma }_{1}^{2}).&&\displaystyle \end{eqnarray*}

The intercept and the slope were allowed to covary. Error terms across time were assumed to be uncorrelated. Time was coded 0 for baseline, 0.25 for post-trial assessment and 1 for termination. Length of therapy and waiting time from trial therapy to treatment start varied. The codings of time were based on an estimation that the average waiting time from trial therapy to post-trial assessment was a third of the time of the length of an average full treatment course.

Second, conditional growth models were estimated to examine whether the growth trajectory of the outcomes (BSI and IIP) differed as a function of having a major unlocking of the unconscious. Unlocking of the unconscious was included as a time-invariant binary coded predictor. This variable reflected whether a patient had a major unlocking of the unconscious anytime during therapy. Patient gender, age, type of treatment resistance, total number of sessions and therapist experience were investigated as potential control predictors. Number of sessions was log10-transformed to improve normality. To determine if a control predictor was to be included in the growth curve models, descriptive analyses were carried out to investigate if there were any BSI or IIP differences at any time point due to the predictor in question. If a significant difference was present, the predictor was included in the analyses. Independent *t*-tests were used to make a decision on binary predictors and one-way ANOVAs were used for continuous predictors converted into quartiles.

Our analytical approach made use of all available data, making this an intention-to-treat analysis. Full information maximum likelihood estimation was used. This form of estimation provides unbiased estimates under the less restrictive assumption of data missing at random (MAR; Mallinckrodt, Clark & David, 2001), which allows the probability of data being missing to be dependent of both outcome variables (e.g., symptom level as measured by the BSI and the IIP) and predictors (Little & Rubin, 2002). To take missing data further into account, we performed additional analyses where informativeness of missing data patterns were investigated as potential confounding factors (Little & Wang, 1996; Hedeker & Gibbons, 1997). As formal dropout was not recorded, we treated post-treatment data missing due to dropout and data missing for other reasons as equivalent.

Within-group effect sizes (Cohen’s *d*) were calculated by dividing the pre-post differences in observed means by the pooled standard deviations (Borenstein et al., 2011).

## Results

### Baseline characteristics and enrollment

There were 1010 participants referred to the clinic during the study. Of those referred, 500 had a trial therapy and did also complete a baseline assessment. Out of these, 412 patients had at least one session after the trial and were then included in the effectiveness evaluation. These patients averaged 41.5 years of age (SD 12.7, data on age missing on 34% of cases) and 59.0% were female. The most common clinically derived DSM-IV diagnoses among the 412 included participants were somatoform disorder (58.7%), anxiety disorders (53.4%), cluster B and C personality disorder (18.2% and 36.7%), and major depression (39.3%). Furthermore, 45.6% had chronic or recurrent headache, 31.3% had pain disorder, 23.5% had irritable bowel syndrome and 14.1% had fibromyalgia. These patients had an average treatment duration of 10.2 sessions (SD 13.3, range 2–100, median 5). A complete description of the flow of participants is given in Fig. 1.

### Data attrition

There were 118 participants who had only one treatment session after the trial therapy. For these, the post-trial assessment was the last measurement. Out of these 118 participants, 83 and 72 completed the BSI and the IIP at post-trial, respectively (i.e., 29.7% and 39.0% missing). Among the 294 participants who had more than two sessions, 168 and 142 participants completed the BSI and the IIP at termination, respectively (i.e., 42.9% and 51.7% missing). Hence, the total attrition at the last assessment point was 39.1% for the BSI and 48.1% for the IIP. This is further illustrated in the flowchart in Fig. 1.

### Therapists and training

Two hundred and twenty cases (53.4%) were treated by 4 graduated psychiatrists or 1 psychologist with a mean of 1374.6 (SD 817.6) total training hours (including didactic training and group videotape supervision). Out of these, 115 cases were treated by a psychiatrist who was considered an expert in ISTDP, with over 2000 h of total training. One hundred and ninety-two cases (46.6%) were treated by 43 trainees with a mean of 229.6 (SD 208.9) total training hours. This latter group was comprised of psychiatry residents (*n* = 29), and a mix of other students and professionals in training (*n* = 14).

### Treatment resistance and major unlocking of the unconscious

Of the 412 included participants, 408 (99.0%) had data on treatment resistance. Out of these, 289 participants (70.8%) met criteria for psychoneurotic and 119 (29.2%) were fragile (*n* = 86) or psychotic (*n* = 33). Furthermore, 153 out of 411 participants (37.2%) had at least one major unlocking of the unconscious throughout treatment (data on unlocking missing for one patient).

### Effects of time (Unconditional growth models)

#### BSI

Results from the unconditional growth model for BSI indicated that there was significant variance in the intercept (symptom level at baseline) and in the slope (rate of decrease of BSI scores over time). The mean BSI trajectory was estimated to start at 1.58 at baseline and to slope downward at a rate of 0.70 BSI units over the course of treatment. The covariance between the intercept and the slope was −0.10, indicating that patients who started with higher BSI scores at baseline had steeper BSI slope trajectories. Effect size (based on observed means) was Cohen’s *d* = 0.87 95% CI [0.71–1.03], indicating a large effect of treatment over time. Means and standard deviations for the raw BSI scores are presented in Table 1.

**Table 1:** Means, SDs and effect sizes (Cohen’s *d*) for the Brief Symptom Inventory (BSI) and the Inventory of Interpersonal Problems (IIP).

	Baseline (SD)	*N*	Termination (SD)	*N*	Mean difference (SD)	*N*	Effect size (95% CI)
**BSI**	1.60 (0.75)	412	0.92 (0.77)	168	0.67 (0.69)	168	0.87 [0.71–1.03]
**IIP**	1.51 (0.64)	390	0.99 (0.68)	144	0.53 (0.63)	142	0.83 [0.64–1.02]

#### IIP

The unconditional growth model used to model IIP indicated significant variance in the intercept and the slope. The covariance between the intercept and the slope was −0.07 (*p* = .07), once again indicating that starting at higher baseline IIP resulted in steeper rate of change. The mean IIP trajectory was estimated to start at 1.50 at baseline and to slope downward at a rate of 0.59 IIP units over the whole treatment. There was a large effect size Cohen’s *d* = 0.83 95% CI [0.64–1.02] over time (based on observed means). Means and standard deviations for the IIP can be found in Table 1.

### Associations of unlocking the unconscious with outcome (Conditional growth models)

#### Control predictors

Type of treatment resistance, therapist experience, patient gender, patient age, and the logarithm of number of treatment sessions were investigated as potential control predictors of outcome. Therapist experience was investigated both by comparing trainees to non-trainees, and by comparing the outcomes of the ISTDP expert to the other therapists. Descriptive analyses of the predictors at all time points revealed that there were BSI differences at baseline with fragile and psychotic patients having larger symptom severity than psychoneurotic patients (independent *t*-test, *t*(406) = 4.3, *p* < .001), with a similar trend on the IIP at baseline (independent *t*(384) = 1.9, *p* = .06) and at post-trial (independent *t*(221) = 1.8, *p* = .08). Furthermore, on IIP there was a difference at termination depending on therapist experience, with the 43 trainees having worse outcome than the six non-trainees (independent *t*(142) = 2.3, *p* < .05) and the expert having better outcome than the other 48 therapists (independent *t*(142) = 2.6, *p* < .05). The ANOVA investigating differences due to number of sessions (log) revealed a difference on the IIP at post-trial (*F*(3, 204) = 3.26, *p* < 0.05). An inspection of the post-trial IIP raw scores indicated that participants who only took part of the trial therapy and the follow-up meeting had lower IIP scores at this time point than those who continued with further therapy. This difference was significant (independent *t*(222) = 2.46, *p* < .05). No other differences due to number of sessions were found at any other time point on neither the BSI nor the IIP (all *F*’s < 1.98, all *p*’s > 0.11). No gender differences at any time point were found (all *t*’s < 1.5, all *p*’s > .14). Similarly, no differences due to age were found (all *F*’s < 1.82, all *p*’s > .14). Based on these findings, we decided to include type of resistance, trainee status, expertise and number of sessions (log) as control predictors.

#### BSI

In an uncontrolled conditional growth model, the variable that measured whether the patient had a major unlocking of the unconscious during therapy (called UNLOCKING in this section) was added at Level 2 as a time-invariant predictor in interaction with the initial status (intercept) and in interaction with time. The latter interaction term was added to determine if, over time, UNLOCKING significantly interacted with the BSI slope, i.e., predicted symptom change during treatment. At this stage of the analysis, the UNLOCKING by time was close to significant (*p* = .08) in the model.

**Figure 2:**
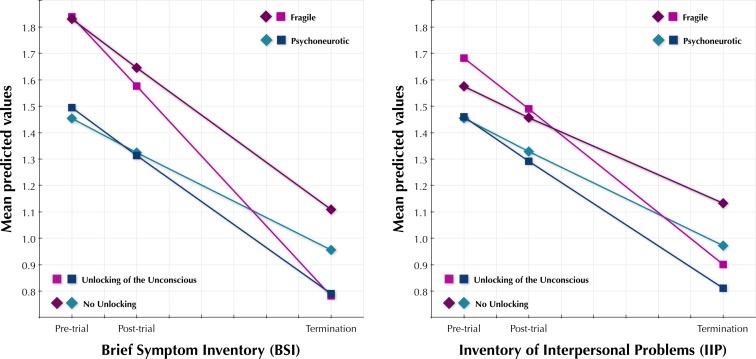
BSI and IIP scores. Estimated means of the Brief Symptom Inventory and the Inventory of Interpersonal Problems at baseline, post-trial and at treatment termination.

The next step carried out was to determine if this association between UNLOCKING and BSI change was still present after accounting for type of treatment resistance, trainee status, expertise and the logarithm of number of treatment sessions (called RESISTANCE, TRAINEE, EXPERT and log(SESSIONS) in this section). Hence, a controlled conditional growth model was carried out. RESISTANCE, TRAINEE, EXPERT and log(SESSIONS) were added as time-invariant predictors in interaction terms with baseline BSI (the intercept) and BSI change (slope). The RESISTANCE and EXPERT by intercept and time were significant, and therefore kept in the model. However, TRAINEE and log(SESSIONS) were not significant neither in interaction with the intercept nor with the slope and therefore left out of the model. Finally, we added information on missingness from the post-trial and post-treatment assessments in interaction with the intercept and with the slope to investigate if data missingness was related to outcome (Little & Wang, 1996; Hedeker & Gibbons, 1997). Neither of these interactions were significant and therefore information on missing data was removed from the model. In the final model (details in Table 2), the UNLOCKING by time interaction was significant (*p* < .05). Estimated means from this model are illustrated in Fig. 2. The equations for the final BSI model were as follows:

Level-1 Model:

\begin{eqnarray*} \displaystyle {Y}_{i j}={\pi {}_{0}}_{i}+{\pi {}_{1}}_{i}\times {T I M E}_{i j}+{\varepsilon }_{i j}&&\displaystyle \end{eqnarray*}

Level-2 Model:

\begin{eqnarray*} \displaystyle \mathrm{Intercept}{:}~{\pi {}_{0}}_{i}={\gamma }_{00}+{\gamma }_{01}\times {\mathit{UNLOCKING}}_{i}+{\gamma }_{02}&&\displaystyle \nonumber\\ \displaystyle \quad \times {\mathit{RESISTANCE}}_{i}+{\gamma }_{03}\times {\mathit{EXPERT}}_{i}+{\zeta \! {}_{0}}_{i}&&\displaystyle \nonumber\\ \displaystyle \mathrm{Slope}{:}~{\pi {}_{1}}_{i}={\gamma }_{10}+{\gamma }_{11 \hspace{0.167em} }\times {\mathit{UNLOCKING}}_{i}+{\gamma }_{12}\times {\mathit{RESISTANCE}}_{i}+{\gamma }_{13}\times {\mathit{EXPERT}}_{i}+{\zeta \! {}_{1}}_{i}&&\displaystyle \nonumber\\ \displaystyle {\varepsilon }_{i j}\sim N(0,{\sigma }_{\varepsilon }^{2}),{\zeta \! {}_{0}}_{i}\sim N(0,{\sigma }_{0}^{2}),{\zeta \! {}_{1}}_{i}\sim N(0,{\sigma }_{1}^{2}).&&\displaystyle \end{eqnarray*}

**Table 2:** Growth models estimating trajectories of change in Brief Symptom Inventory (BSI) and Inventory of Interpersonal Problems (IIP) from baseline to treatment termination.

			BSI		IIP	
		Parameter	Unconditional growth model	Final controlled growth model	Unconditional growth model	Final controlled growth model
**Fixed effects**						
Initial status, *π*_0*i*_	Intercept	*γ*_00_	1.58^***^	1.47^***^	1.50^***^	1.45^***^
	Unlocking	*γ*_01_		0.04		0.03
	Resistance	*γ*_02_		0.37^***^		0.15^*^
	Expertise	*γ*_03_		−0.04		
Rate of change, *π*_1*i*_	Intercept	*γ*_10_	−0.70^***^	−0.46^***^	−0.59^***^	−0.50^***^
	Unlocking	*γ*_11_		−0.21^*^		−0.20^*^
	Resistance	*γ*_12_		−0.22^*^		
	Expertise	*γ*_13_		−0.22^*^		
**Variance components**						
Level 1	Within-person	${\sigma }_{\varepsilon }^{2}$	0.16^***^	0.15^***^	0.13^***^	0.13^***^
Level 2	In initial status	${\sigma }_{0}^{2}$	0.42^***^	0.40^***^	0.31^***^	0.31^***^
	In rate of change	${\sigma }_{1}^{2}$	0.17^**^	0.15^**^	0.14^**^	0.13^*^
	Covariance	*σ*_01_	−0.10^*^	−0.08^*^	−0.07^*tr*^	−0.06

**Notes.**

*tr* trend

* *p* < .05.

** *p* < .01.

*** *p* < .001.

#### IIP

Initially UNLOCKING was added as a predictor and was close to significant (*p* = .07) in interaction with time. RESISTANCE was added and was significant in interaction with the intercept, but not with the slope. Hence, the RESISTANCE times slope interaction was left out of the final model. Neither TRAINEE, EXPERT nor log(SESSIONS) were significant in interaction with the intercept or the slope, and therefore not included in the model. In the final model, the UNLOCKING by time interaction was significant (*p* < .05). As in the BSI model, information on missingness was not significant and was therefore not included. Results from the IIP model are illustrated in Fig. 2. Details of the final model can be found in Table 2 and in the equations below:

Level-1 Model:

\begin{eqnarray*} \displaystyle {Y}_{i j}={\pi {}_{0}}_{i}+{\pi {}_{1}}_{i}\times {\mathit{TIME}}_{i j}+{\varepsilon }_{i j}&&\displaystyle \end{eqnarray*}

Level-2 Model:

\begin{eqnarray*} \displaystyle \mathrm{Intercept}{:}~{\pi {}_{0}}_{i}={\gamma }_{00}+{\gamma }_{01 \hspace{0.167em} }\times {\mathit{UNLOCKING}}_{i}+{\gamma }_{02 \hspace{0.167em} }\times {\mathit{RESISTANCE}}_{i}+{\zeta \! {}_{0}}_{i}&&\displaystyle \end{eqnarray*}

\begin{eqnarray*} \displaystyle \mathrm{Slope}{:}~{\pi {}_{1}}_{i}={\gamma }_{10}+{\gamma }_{11 \hspace{0.167em} }\times {\mathit{UNLOCKING}}_{i}+{\zeta \! {}_{1}}_{i}&&\displaystyle \nonumber\\ \displaystyle {\varepsilon }_{i j}\sim N(0,{\sigma }_{\varepsilon }^{2}),{\zeta \! {}_{0}}_{i}\sim N(0,{\sigma }_{0}^{2}),{\zeta \! {}_{1}}_{i}\sim N(0,{\sigma }_{1}^{2}).&&\displaystyle \end{eqnarray*}

## Discussion

In this paper, we have established the effectiveness of Davanloo’s Intensive Short-Term Dynamic Psychotherapy when delivered by a range of therapists in a clinically representative setting to a large number of patients. Importantly, the present study was carried out in a tertiary care clinic, which is assumed to have implied a more treatment-resistant patient sample than those present in standard clinics. This study adds to the overall evidence base of ISTDP in particular, but also to that of psychodynamic psychotherapy in general. In a recent meta-analysis on randomized controlled trials of psychodynamic psychotherapy (Town et al., 2012a), the overall within-group effect size was estimated to Cohen’s *d* = 1.01 95% CI [0.86–1.16]. Furthermore, a study evaluating the overall effectiveness of psychotherapy for depression among 5,704 clinical patients in a managed care setting (Minami et al., 2008) estimated the effect size to be *d* = 0.75 95% CI [0.72–0.77]. Hence, the effects observed in this study (effect sizes Cohen’s *d* = 0.87 (95% CI [0.71–1.03] and *d* = 0.83 95% CI [0.64–1.02] for the BSI and the IIP, respectively), seem to be comparable both to effects of psychodynamic psychotherapy when provided in RCTs and to that of psychotherapy in general when provided in clinical practice.

In this study, we also showed that a major unlocking of the unconscious during therapy predicted better treatment outcome. This result validates a fundamental assertion in Davanloo’s ISTDP and is to our knowledge the first study to systematically reproduce those assertions in a large-scale patient sample (Town, Abbass & Bernier, 2013). Moreover, this finding is in line with research suggesting that affective experience during psychodynamic psychotherapy may enhance treatment effectiveness (Whelton, 2004; Diener, Hilsenroth & Weinberger, 2007; Diener & Hilsenroth, 2009; Salvadori, 2010). Importantly, strong emotional breakthroughs in ISTDP are assumed to be dependent on the therapist’s systematic challenge to resistance in session. Hence, our findings in this paper are likely to parallel recent research that have highlighted a relationship between affective arousal and preceding active therapist confrontation (Town et al., 2012b).

In the investigation of the associations of unlocking with outcome, we included a number of control predictors including type of treatment resistance. The association of type of treatment resistance with outcome can be interpreted as a separate effect, after controlling for the main predictor of interest (unlocking the unconscious). Patients classified as fragile or psychotic had more symptom severity at baseline than those who were psychoneurotic. This is in line with psychodynamic theory suggesting that individuals with access to primitive defenses (fragile or psychotic) have been exposed to more adverse and/or persistent childhood experiences: this on its own is known to be associated with higher symptom severity in later life (Felitti et al., 1998; Edwards et al., 2003). Furthermore, we found that fragile patients had (compared to psychoneurotic patients) steeper rate of change on the BSI, while there was no such difference in slope on the IIP. This suggests that, to understand the larger degree of change in reduced symptom distress seen in fragile patients, it may be necessary to look beyond the concept of achieving a major unlocking of the unconscious to other therapeutic variables. As described above, ISTDP for fragile patients typically involves building capacity for tolerating emotional experiences (Davanloo, 1990; Whittemore, 1996; Abbass & Bechard, 2007). Hence, results from this study could indicate that this capacity-building format may be a key intervention when working with patients with primitive defenses, character pathology and with a high symptom burden. Furthermore, we replicated previous findings showing that the rate of change on the IIP is associated with unlocking the unconscious, but not with any other predictor (Town, Abbass & Bernier, 2013). Given the fact that change on the IIP may reflect character change rather than symptom change, the typical treatment course in this study (median 5 sessions, 10.2 on average) may not have been long enough to achieve major changes on the IIP. Thus, if treatment courses had been longer, we could possibly have expected a larger variation among IIP slopes, which then potentially could be explained by other predictors than unlocking the unconscious. This could parallel the claim by Davanloo that for major character change to take place, 20–40 sessions of ISTDP may be needed for psychoneurotic patients and 60–80 for those with fragile character structure (Davanloo, 2005).

Whether experienced therapists perform better than less experienced is a debated topic in the psychotherapy research community, with limited literature suggesting that more training enhance treatment effectiveness (Hattie, Sharpley & Rogers, 1984; Wampold & Brown, 2005). This is in line with our findings that the group of professional therapists (vs. trainees) with far more training hours did not seem to conduct more effective treatments. It also parallels previous research on the effectiveness of ISTDP when carried out by psychiatry residents that indicated reductions in both symptoms and health care costs (Abbass, 2004; Abbass et al., 2013). However, we found that a single therapist, considered an expert in ISTDP, had better outcomes than the other therapists, on one of the outcome measures. This is indeed interesting as expertise in psychotherapy is largely unexplored (Tracey et al., 2014). Further research on expert performance in ISTDP is warranted based on these findings. To extend the current findings, this may include examination of therapists’ competence as measured by the quality of the unlocking of the unconscious.

Strengths of this study include that the treatment was tested for a large-scale sample of 412 patients, that therapists were trained and supervised by a highly experienced ISTDP teacher, and that data analyses were conducted using advanced statistical methods capable of making use of all available data. A further strength is that several elements contribute to the generalizability of the findings from this study: the treatment was provided in a clinically representative setting by a large number of therapists with different levels of experience, all patients were recruited by referral from professionals rather than advertisement, and very broad selection criteria were applied that allowed a mixed sample of patients to be included.

However, there are limitations of this research that need to be addressed. First, a substantial number of patients did not have a post-treatment assessment (missing because of dropout or other reasons, 39.1% on the BSI, 48.1% on the IIP). We addressed this limitation by using data-analytic procedures that have been shown to be valid under the assumption that the data was missing at random (MAR). This allows the probability of data being missing to be dependent upon both outcome variables (e.g., symptom severity) and predictors (Little & Rubin, 2002). Furthermore, we conducted additional pattern mixture models to take data missingness into account (Little & Wang, 1996; Hedeker & Gibbons, 1997). Despite the use of these data-analytic procedures, we cannot exclude the possibility that the results from this study have been affected by the amount of missing data. The actual rate of dropout in this study was difficult to determine also because many patients came from far away and only attended a few sessions for travel reasons. Importantly though, meta-analytic estimations of the number of dropouts have found similar rates as in this study. An investigation of 125 psychotherapy outcome studies estimated the mean dropout rate to 46.9% (Wierzbicki & Pekarik, 1993). More recently, a meta-analysis of 34 non-randomized effectiveness studies on outpatient cognitive behavioral therapy for depression found that 42.0% of patients who received individual therapy dropped out (Hans & Hiller, 2013). Our rates of missing data and/or dropout also parallel a recent large-scale effectiveness evaluation of psychodynamic psychotherapy for depression in which 45.2% of the participants dropped out or were lost to assessment (Driessen et al., 2013). A second limitation in this study was the fact that the ratings of rise in the complex transference feelings and degree and type of treatment resistance were conducted using non-validated instruments. Measurements of these constructs in this study were carried out by a highly experienced ISTDP clinician and teacher. We have because of the experience of the coder assumed adequate validity and reliability of these measurements. However, this procedure might have caused a measurement bias due to the theoretical orientation of the ISTDP teacher. In conclusion, this limitation highlights the need to develop psychometrically sound and reproducible procedures to measure the constructs proposed by Davanloo (1990), Davanloo (2000), Davanloo (2001) and Davanloo (2005). Third, the results in this study on unlocking in relation to outcome are correlational, i.e., we have not established a causal effect. Hence, we cannot exclude a possible reversed causal effect, that symptom relief on its own resulted in emotional breakthroughs. While we consider this as unlikely due to the fact that a major unlocking is assumed to be the result of the breakdown of treatment resistance after systematic challenge, we acknowledge the need for future research that establish a causal relationship between outcome and in-session events in ISTDP. Fourth, data from self-report measures was collected only at three time points instead of weekly assessments. While a weekly collection might not have been possible to carry out within the current routine care setting, it would have contributed to a better estimate of the treatment effect over time. A related concern is that the research project did not record the exact times from baseline to post-trial and from post-trial to termination. Instead, estimated averages for these time periods times were used. We acknowledge this lack of a more precise coding of time as a limitation of the study. A final limitation is that the lack of control condition and the non-randomized effectiveness design of this study did not permit us to control for gains due to time passing (e.g., spontaneous gains and gains from other treatments received). Nor was there any extended baseline assessment phase that could have been used to estimate the effect of time before treatment started. Hence, we cannot exclude the possibility that the treatment effects observed in this study were due to other factors than the treatment received. However, this is unlikely given the short treatment duration and the likely chronicity of patient problems referred to this service.

Based on the findings from this study, further research on ISTDP is warranted. We see a continued need for systematic investigation of the efficacy of ISTDP for specific diagnostic groups using randomized controlled trials. Such research could also include assessments of in-session events such as rise in complex transference feelings, that would enable more detailed examinations of the relation between events such as the major unlocking of the unconscious and outcome. Furthermore, standardized ratings of rise in the complex transference feelings and type and degree of resistance should be developed for future research on ISTDP, to enable further knowledge of the mechanisms of change in ISTDP. A guide for such ratings is in development (JM Town & A Abbass, 2014, unpublished data).

## Conclusions

This study shows that Davanloo’s Intensive Short-Term Dynamic Psychotherapy is an effective treatment when carried out in tertiary care, when delivered by therapists with a range of experience. Furthermore, this study provides support for the key assumption in Davanloo’s ISTDP in that unlocking the unconscious is associated with enhanced treatment outcome. Further research on the effectiveness of ISTDP and its working mechanisms is warranted.

## Supplemental Information

10.7717/peerj.548/supp-1 Supplemental Information 1 CONSORT checklist

## Additional Information and Declarations

### Clinical Trial Registration

The following information was supplied regarding Clinical Trial registration: ClinicalTrials.gov: NCT01924715.
